# Emerging roles of natural killer cell ligands—HLA-E, HLA-F, HLA-G, MICA, and MICB—in *in vitro* fertilization outcomes

**DOI:** 10.3389/fgene.2025.1661511

**Published:** 2025-11-13

**Authors:** Itta Krishna Chaaithanya, Raja Rajalingam

**Affiliations:** 1 Immunogenetics and Transplantation Laboratory (ITL), University of California, San Francisco (UCSF), San Francisco, CA, United States; 2 Department of Molecular Immunology & Microbiology, Indian Council of Medical Research-National Institute for Research in Reproductive and Child Health (ICMR-NIRRCH), Mumbai, India; 3 Academy of Scientific and Innovative Research (AcSIR), Ghaziabad, Uttar Pradesh, India

**Keywords:** infertility, *in vitro* fertilization (IVF), non-classical HLA, uterine NK cells, immune tolerance, implantation, reproductive immunogenetics

## Abstract

Infertility affects approximately one in six individuals globally and represents a complex public health concern influenced by a range of biological, environmental, and socioeconomic factors. *In vitro* fertilization (IVF) has emerged as a pivotal assisted reproductive technology (ART), yet its success is often hindered by recurrent implantation failure (RIF) and hypertensive complications such as preeclampsia (PE). Recent research highlights the critical role of the immune system particularly non-classical Human Leukocyte Antigen (HLA) class I molecules (HLA-G, HLA-E, HLA-F) and MHC class I chain-related proteins (MICA/B) in modulating maternal-fetal tolerance and determining IVF outcomes. This review synthesizes emerging evidence on the structure, expression, receptor interactions, and polymorphisms of these molecules, emphasizing their roles in embryo implantation, immune modulation, and pregnancy maintenance. Soluble HLA-G (sHLA-G) has shown promise as a biomarker for embryo viability, while variations in KIR–HLA interactions and polymorphisms in non-classical HLA genes have been linked to RIF and adverse reproductive outcomes. Despite promising findings, routine clinical testing of these markers remains limited due to methodological inconsistencies, lack of large-scale validation, and the multifactorial nature of implantation. Future research priorities include functional genomics, standardized diagnostic assays, AI-driven predictive tools, and translational trials of immunomodulatory therapies. Understanding the immunogenetic landscape offers new avenues for personalized reproductive care and improved IVF success rates.

## 1 Introduction

Infertility, defined as the inability to achieve pregnancy after 12 months of regular, unprotected sexual intercourse, represents a significant and growing global public health issue, affecting approximately one in six individuals worldwide (WHO, 2024). Its causes are multifactorial: female and male factors each account for around 35% of cases, while combined factors contribute to 20%, and unexplained infertility to the remaining 10%. Prevalence rates vary regionally, with the Western Pacific reporting the highest at 23.2%, and the Eastern Mediterranean the lowest at 10.7%. Data from Southeast Asia remains limited and inconclusive, though the actual prevalence is likely underestimated (WHO, 2023).

Many causes of infertility are preventable, including those related to untreated infections, environmental exposures, and modifiable lifestyle factors. Among current treatment options, *in vitro* fertilization (IVF) is one of the most effective and widely utilized forms of assisted reproductive technology (ART). IVF involves ovarian stimulation, oocyte retrieval, fertilization either via conventional insemination or intracytoplasmic sperm injection (ICSI) embryo culture, and uterine transfer. Hormonal support enhances endometrial receptivity, and a pregnancy test is conducted approximately 2 weeks post-transfer to assess implantation success (Beresniak et al., 2023). Success rates vary significantly by age, with live birth rates around 49.7% in women under 35, and declining markedly in older age groups (Somigliana et al., 2023). However, IVF remains financially and emotionally demanding, often not covered by insurance, and access remains limited in low-resource and rural areas (Zhou et al., 2019; Mikhael et al., 2021). Ethnic disparities also exist; for example, Indian women have been reported to have lower IVF success rates compared to Caucasian women, potentially due to differences in embryo quality (Lashen et al., 1999; Shahine et al., 2009).

A major clinical challenge in ART is Recurrent Implantation Failure (RIF), generally defined as the failure to achieve a clinical pregnancy after three or more transfers of high-quality embryos. RIF affects an estimated 10%–15% of women undergoing IVF (Simon and Laufer, 2012; Bashiri et al., 2018; Mao et al., 2023). Factors contributing to RIF include advanced maternal age, poor gamete quality, chromosomal abnormalities, suboptimal uterine receptivity, and immunological dysfunction at the maternal–fetal interface. Structural uterine anomalies such as fibroids, endometriosis, hydrosalpinx, and endometrial polyps further hinder successful implantation. Additionally, lifestyle factors such as obesity, smoking, and alcohol use have been shown to negatively impact IVF outcomes (Cakmak and Taylor, 2011; Das and Holzer, 2012; Penzias, 2012; Coughlan et al., 2014). Recent studies also highlight the role of genetic polymorphisms in reproductive outcomes (Zhu et al., 2018; Lee et al., 2019).

Another serious complication of IVF pregnancies is preeclampsia (PE) a hypertensive disorder characterized by elevated blood pressure and multisystem involvement, typically occurring after 20 weeks of gestation. IVF-conceived pregnancies, particularly those using donor oocytes, are associated with a substantially higher risk of PE. Women using donor eggs are four to five times more likely to develop PE compared to those with spontaneous pregnancies, and two to three times more likely than those using autologous oocytes (Wang et al., 2020; Keukens et al., 2022). The etiology is multifactorial, involving maternal characteristics, ART-related procedures, and nutritional and immunological factors (Berlinska et al., 2021; Kinshella et al., 2022; Maducolil et al., 2022).

The success of IVF depends on a highly coordinated interplay of endocrine, genetic, and immunological factors. Among these, the Human Leukocyte Antigen (HLA) system plays a pivotal role in establishing maternal immune tolerance to the semi-allogenic embryo. Both classical (e.g., HLA-C) and non-classical HLA class I molecules (e.g., HLA-G, HLA-E, HLA-F), along with MHC class I chain-related molecules (MICA/B), are implicated in implantation success and pregnancy maintenance (Geraghty et al., 1990; Shaikly et al., 2008; Sipak-Szmigiel et al., 2009; Guo et al., 2013; Zhu et al., 2018; Lee et al., 2019; Wilczyńska et al., 2020; Piekarska et al., 2021). While classical HLA genes have been extensively studied in reproductive immunology (Hiby et al., 2010; Lashley et al., 2014; Moffett et al., 2016; Alecsandru et al., 2020), the roles of non-classical HLA class I genes and MICA/B in IVF outcomes remain relatively underexplored.

This review provides an in-depth analysis of the emerging roles of non-classical HLA class I genes and MIC genes in IVF success. We examine their expression patterns, structural characteristics, receptor interactions, and ligand polymorphisms, with special emphasis on their roles in embryo implantation, immune modulation, and pregnancy outcome. Understanding these immunogenetic factors may offer novel insights for improving ART outcomes and developing predictive biomarkers for implantation and pregnancy success.

## 2 Immunomodulatory roles of non-classical HLA and MIC molecules

Non-classical HLA class I molecules including HLA-G, HLA-E, HLA-F, and the MICA/B family exhibit distinct expression patterns, limited genetic polymorphism, and specialized immunomodulatory functions that differentiate them from classical HLA class I molecules. While classical HLA class I molecules primarily regulate cytotoxic T lymphocyte responses, non-classical HLA and MIC molecules predominantly interact with natural killer (NK) cells, modulating their activity to promote immune tolerance. These interactions are essential for establishing and maintaining maternal–fetal immune tolerance, particularly during early pregnancy and embryo implantation. Collectively, non-classical HLA and MIC molecules coordinate a finely tuned immunological environment critical for successful implantation and pregnancy progression. Increasing evidence links their genetic diversity and functional expression to reproductive outcomes, positioning them as emerging biomarkers and potential therapeutic targets in the context of ART success.

## 3 HLA-G and reproductive immunology in IVF

### 3.1 HLA-G: structure, function, and expression

HLA-G is a nonclassical HLA class I molecule that plays a pivotal role in immune regulation, particularly in pregnancy. HLA-G exerts its regulatory function by engaging inhibitory receptors such as ILT2, ILT4, and KIR2DL4 present on NK cells, T cells, and antigen-presenting cells. These interactions dampen cytotoxic responses, influence cytokine release, and foster a tolerogenic immune environment critical for maternal–fetal tolerance. Unlike classical HLA class I molecules (HLA-A, -B, -C), HLA-G exhibits limited polymorphism, reflecting its conserved immunological function. As of September 2025, 194 HLA-G alleles have been identified, of which 61 encode functional proteins and 6 are null alleles (Robinson et al., 2024). Structurally, the HLA-G gene includes eight exons and seven introns. Exons 2–4 encode the extracellular domains involved in antigen presentation and receptor binding, while exon 8 forms the 3′untranslated region (3′UTR), crucial for regulating mRNA stability and translation. HLA-G typically forms a non-covalent complex with β2-microglobulin, essential for its stability and function.

**FIGURE 1:**
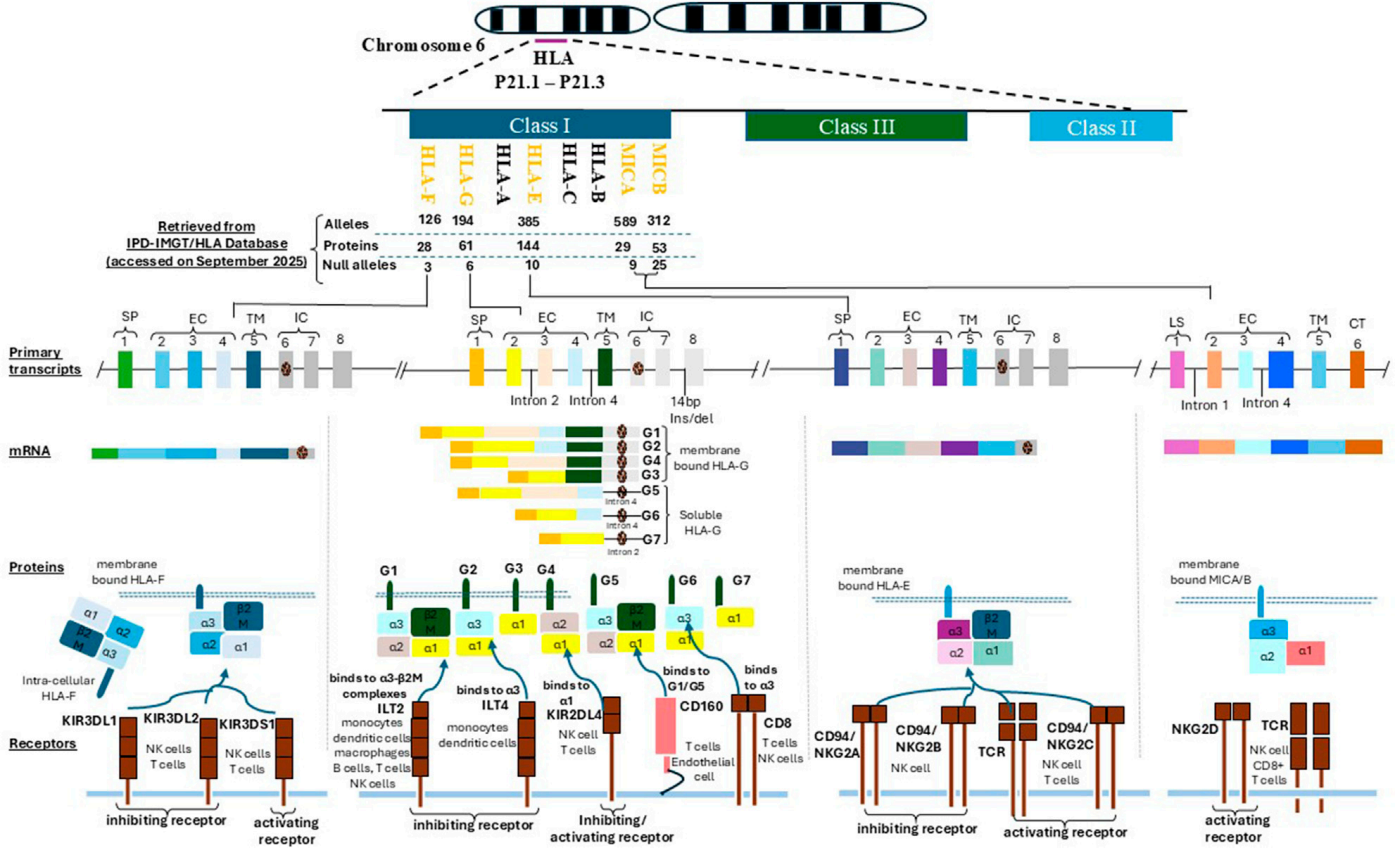
This figure illustrates the genomic organization and immunological functions of non-classical HLA class I genes HLA-G, HLA-F, HLA-E, MICA, and MICB located within the chromosome 6p21.1–p21.3 region. It highlights their structural domains, protein isoforms, and receptor interactions that underpin their roles in immune regulation during implantation and pregnancy. Each HLA molecule shares a conserved architecture comprising a signal peptide (SP), three extracellular domains (α1, α2, α3) responsible for ligand binding and receptor interaction, a transmembrane domain (TM) anchoring the molecule to the cell surface, and an intracellular domain (IC) involved in intracellular signaling. HLA-G, HLA-F, and HLA-E are primarily expressed as membrane-bound molecules, with HLA-G uniquely generating both membrane-bound (e.g., G1) and soluble isoforms (e.g., G5) through alternative splicing. MICA and MICB are induced under cellular stress conditions (e.g., infection, oxidative stress, or malignant transformation) and are typically expressed on the cell surface of epithelial and endothelial cells. Receptor interactions are pivotal to their immunomodulatory functions. HLA-G engages ILT2 (LILRB1) and ILT4 (LILRB2) via its α3 domain and interacts with KIR2DL4 through its α1 domain, leading to inhibition of NK and T cell cytotoxicity. Soluble isoforms of HLA-G also bind CD160, influencing T cell and endothelial cell responses. HLA-E binds both the inhibitory receptor CD94/NKG2A and the activating receptor CD94/NKG2C, modulating NK and CD8^+^ T cell activity in a peptide-dependent manner. HLA-F, which may function in both open conformer and peptide-bound states, interacts with KIR3DL2 (inhibitory) and KIR3DS1 (activating) receptors on NK and T cells. MICA and MICB act as ligands for the activating receptor NKG2D, expressed on NK cells, CD8^+^ αβ T cells, and γδ T cells. This interaction promotes cytotoxic immune responses, contributing to immune surveillance and implantation-related inflammation. These molecular interactions support the establishment of maternal–fetal immune tolerance, regulate decidual NK cell activity, and influence reproductive outcomes in both natural and assisted conception.

At the maternal–fetal interface, HLA-G binds to inhibitory receptors on immune cells ILT2 and ILT4 (monocytes, dendritic cells, T cells), and KIR2DL4 (uterine NK cells) (Figure 1). This interaction suppresses cytotoxicity and promotes immune tolerance. Additionally, HLA-G stimulates anti-inflammatory cytokines (e.g., IL-10, TGF-β) and angiogenic factors (e.g., Vascular Endothelial Growth Factor (VEGF)), facilitating placental vascularization and spiral artery remodeling. Notably, HLA-G has been linked to senescence-like changes in trophoblast and endothelial cells, aiding early pregnancy adaptations (LeMaoult and Yan, 2021). Single-cell analyses of decidual tissue reveal dynamic regulation of HLA-G across trophoblast subtypes. Reduced HLA-G expression is associated with adverse outcomes, including PE, RIF, and miscarriage (Zhou et al., 2019). Thus, HLA-G serves as a key mediator of immune tolerance, vascular remodeling, and successful implantation.

### 3.2 Soluble HLA-G and its role in IVF success

Soluble forms of HLA-G (sHLA-G), mainly the HLA-G5 and HLA-G6 isoforms, are secreted into extracellular fluids and play critical roles in early embryo–maternal communication. These isoforms are detectable in follicular fluid, embryo culture media, maternal serum, and uterine secretions, and are believed to promote implantation and maternal immune adaptation during assisted reproduction.

Pioneering studies by Jurisicova and colleagues (1996) (Jurisicova et al., 1996) demonstrated sHLA-G expression from the 2-cell to blastocyst stage. Elevated sHLA-G levels in embryo culture media have since been linked to increased implantation rates and pregnancy success in IVF (Fuzzi et al., 2002). Clinical studies have shown that low maternal sHLA-G correlates with early pregnancy loss (Pfeiffer et al., 2000), while higher levels are associated with ongoing pregnancies. A meta-analysis of over 6,000 IVF cases confirmed that embryos secreting sHLA-G were significantly more likely to implant and result in clinical pregnancy (Niu et al., 2017). Furthermore, combining sHLA-G quantification with embryo morphology scores enhances predictive accuracy (Kotze et al., 2010; Kotze et al., 2013). sHLA-G predominantly localizes to the trophectoderm, supporting its role in embryo–endometrium interaction (Shaikly et al., 2008).

Despite these encouraging findings, the clinical utility of sHLA-G remains contested. Several studies investigating RIF failed to confirm consistent associations between sHLA-G levels and reproductive outcomes (Vani et al., 2021; Hu et al., 2022). These discrepancies may stem from differences in assay platforms, thresholds used, and sampling windows across studies. These disparities underscore a critical need for harmonized protocols before sHLA-G can be considered a reliable biomarker.

Importantly, the absence of sHLA-G may reflect intrinsic embryonic abnormalities rather than maternal immune defects (Criscuoli et al., 2005). New technologies, including Luminex-based assays and AI-driven embryo selection tools, are increasingly integrating sHLA-G data to enhance IVF personalization (Tang et al., 2024). Although promising, these innovations require international harmonization and large-scale validation to ensure their clinical utility. Despite ongoing challenges, sHLA-G remains a promising non-invasive biomarker for embryo competence and IVF success, pending further standardization and validation. Future studies should focus on validating optimal sHLA-G thresholds in diverse populations and IVF settings to support its implementation in routine clinical decision-making.

### 3.3 HLA-G polymorphisms and their influence on IVF outcomes

Genetic variation within HLA-G, particularly in the 3′UTR, critically affects its expression and reproductive outcomes. The most studied variant is the 14-base pair (bp) insertion/deletion (ins/del) polymorphism (rs371194629). The insertion allele is associated with reduced mRNA stability and lower sHLA-G levels, contributing to impaired immune tolerance and increased risk of implantation failure and miscarriage (Hviid et al., 2004; Costa et al., 2016). Studies across populations have linked the ins allele to recurrent IVF failure (Sipak-Szmigiel et al., 2009; Fan et al., 2017). Conversely, the del allele correlates with enhanced HLA-G expression and better reproductive outcomes. This suggests that host genetic background could be a major determinant of IVF success, yet allele frequency differences across populations may partly explain variability in findings.

Additional polymorphisms, such as rs1063320 (+3142C>G) and rs1632947 (−964G>A), influence mRNA decay and transcription, respectively. These variants, along with others like rs1233334 and rs9380142, have been variably linked to IVF success (Hu et al., 2022), though findings remain inconsistent across ethnicities and study designs. Such heterogeneity underscores the need for larger, ethnically diverse cohorts and standardized genotyping methods to draw robust conclusions. Notably, some polymorphisms initially associated with infertility (e.g., HLA-G*01:03) have shown different trends in broader populations, suggesting complex gene environment interactions (Costa et al., 2016). Interestingly, endometrial sHLA-G levels do not consistently differ by 14-bp genotype or RIF status (Papúchová et al., 2022), highlighting the dominant role of embryo-derived HLA-G. However, higher endometrial expression during the implantation window may support pregnancy success (Kofod et al., 2017). This observation implies that maternal genotype may not fully reflect the local immune-tolerant environment, reinforcing the importance of embryo competence in reproductive immunology. HLA-G haplotypes, such as UTR-4, which combines favorable polymorphisms, have been linked to shorter time to pregnancy (Nilsson et al., 2020). Still, prospective studies are needed to assess whether haplotype-based screening can meaningfully inform IVF strategies. Next-generation sequencing is advancing our understanding of HLA-G’s role in IVF, though clinical testing for polymorphisms remains investigational. Translating these genetic insights into clinical practice will require rigorous validation and cost-effectiveness evaluation.

### 3.4 HLA-G receptors in maternal immune regulation

#### 3.4.1 KIR2DL4: a specialized NK cell receptor

KIR2DL4, an unusual member of the KIR family, is primarily expressed in uterine NK (uNK) cells. Unlike typical surface-bound KIRs, KIR2DL4 functions via intracellular signaling after endocytosis of HLA-G. Its engagement triggers the NF-κB pathway and induces a cytokine-rich, non-cytolytic phenotype that supports decidualization and vascular remodeling (Rajagopalan, 2010).

#### 3.4.2 ILT2 and ILT4: broad immunosuppressive pathways

ILT2 (LILRB1) and ILT4 (LILRB2) are expressed on monocytes, dendritic cells, and NK cells. Their binding to the α3 domain of HLA-G transduces inhibitory signals via ITIM motifs, dampening immune responses and promoting tolerance. ILT2 engagement reduces cytotoxicity in decidual NK and T cells, while ILT4 induces a regulatory macrophage phenotype (Mao et al., 2023). Soluble HLA-G can also bind and be internalized by these receptors, sustaining long-term immunomodulation and potentially contributing to maternal immune memory in subsequent pregnancies.

#### 3.4.3 Genetic and clinical implications

Polymorphisms in the KIR genes and HLA-G genes play a crucial role in regulating receptor–ligand interactions that control maternal immune responses during pregnancy. KIR genes are highly polymorphic and are broadly grouped into two haplotypes: KIR A, which contains mostly inhibitory receptors, and KIR B, which includes a greater number of activating receptors. For example, mothers with the KIR AA genotype—dominated by inhibitory KIRs—who carry fetuses with the HLA-C2 ligand are at increased risk of pregnancy complications, such as PE and recurrent miscarriage (RM) (Hiby et al., 2010). These KIR–HLA interactions influence the activity of maternal uNK cells, which are critical for placental development. Ongoing research is exploring these immune pathways as potential biomarkers and therapeutic targets, with the goal of improving outcomes in assisted reproduction, particularly in cases of immune-mediated infertility.

### 3.5 KIR genotypes and IVF outcomes: maternal and paternal roles

KIR genes, highly polymorphic and categorized into A (inhibitory) and B (activating) haplotypes, significantly affect reproductive success.

#### 3.5.1 Maternal KIR genotype and implantation

Women with the KIR AA genotype, when paired with a fetus carrying HLA-C2, are at increased risk of implantation failure and miscarriage due to excessive inhibition of uNK cells (Hiby et al., 2010; Maftei et al., 2023). This suppresses angiogenesis and trophoblast invasion, essential for successful pregnancy.

#### 3.5.2 Activating KIRs and improved IVF outcomes

Activating KIRs, particularly KIR2DS1, promotes NK cell-mediated secretion of cytokines such as VEGF and Granulocyte-Macrophage Colony-Stimulating Factor (GM-CSF), enhancing implantation. Women with KIR B haplotypes and fetal HLA-C2 show higher pregnancy rates (Wilczyńska et al., 2020). Emerging evidence also suggests paternal KIR genotype may influence semen quality and fertilization capacity (Costa et al., 2012).

#### 3.5.3 Personalizing IVF based on KIR-HLA interactions

KIR and HLA-C genotyping is being explored as a tool for IVF personalization, especially in RIF or unexplained infertility. Epigenetic regulation and the uterine environment further modulate these effects, underscoring the complexity of NK cell activity in implantation (Hiby et al., 2008).

### 3.6 Immunomodulatory therapies for KIR AA–Related implantation failure

For women with the KIR AA genotype, immunomodulatory interventions offer new therapeutic options in IVF.

#### 3.6.1 Corticosteroids and tacrolimus

A Romanian study (Seles et al., 2024) found that combining prednisone and tacrolimus improved IVF outcomes in KIR AA women. Prednisone reduces inflammation, while tacrolimus suppresses T-cell activity and promotes a Th2-dominant, tolerogenic environment favorable to implantation (Nakagawa and Sugiyama, 2024).

#### 3.6.2 Granulocyte colony-stimulating factor (G-CSF) therapy

G-CSF has shown promise in enhancing endometrial receptivity and immune cell recruitment. Trials have reported improved implantation rates in RIF patients treated with G-CSF, especially those lacking activating KIR genes (Scarpellini and Sbracia, 2009).

#### 3.6.3 Precision immunotherapy in reproductive medicine

Personalized immunotherapy based on KIR and HLA genotypes is gaining traction. Pilot studies advocate algorithm-based protocols that tailor treatments such as corticosteroids, G-CSF, tacrolimus, or IVIG to individual immunogenetic profiles (Garrido-Giménez et al., 2023). Larger trials are needed to validate these approaches, standardize regimens, and assess long-term safety and efficacy.

## 4 HLA-E and its ligands in IVF

### 4.1 Structure, expression, and immunological function

HLA-E is a nonclassical HLA class I molecule first characterized by Koller et al., in 1988. Unlike the highly polymorphic classical HLA-Ia molecules (HLA-A, -B, -C), HLA-E exhibits limited genetic variability and a highly specialized immunological role. Structurally, HLA-E forms a heterodimer composed of a membrane-bound α heavy chain (∼45 kDa) and a β2-microglobulin light chain. Its gene consists of seven exons: exons 1–4 encode the extracellular domains (α1, α2, α3), exon 5 the transmembrane region, and exons six and 7 the cytoplasmic tail. Despite this genomic structure, exons 7 and 8 are typically excluded from the mature mRNA transcript, resulting in a shortened cytoplasmic tail (Arnaiz-Villena et al., 2022) (Figure 1).

HLA-E expression is widespread across tissues and tightly regulated at the cell surface, requiring peptides derived from the leader sequences of other HLA class I molecules, especially HLA-G, with which it shares strong functional synergy. These leader peptides stabilize HLA-E at the surface and facilitate its interactions with immune receptors. Due to its restricted peptide repertoire and conserved anchor residues, HLA-E’s function is more immunoregulatory than antigen-presenting (Walpole et al., 2010).

As of September 2025, the IPD-IMGT/HLA Database lists 385 HLA-E alleles, including 144 protein-coding and 10 null variants (Robinson et al., 2024). Among these, HLA-E*01:01 and HLA-E*01:03 are the two most prevalent alleles, found at roughly equal frequencies across ethnic groups, suggesting balancing selection that preserves both high- and low-expression variants (Grimsley and Ober, 1997; Tamouza et al., 2007). Immunologically, HLA-E exerts dual roles in innate and adaptive immunity.

- In innate immunity, HLA-E binds to CD94/NKG2A inhibitory receptors on NK cells, suppressing cytotoxic activity and promoting immune tolerance, especially in immune-privileged sites like the maternal–fetal interface.
- In adaptive immunity, HLA-E can present noncanonical peptides to CD8^+^ T cells, contributing to antiviral and alloimmune surveillance (Sullivan et al., 2008; Persson et al., 2017).

Additionally, HLA-E interacts with a range of immunomodulatory receptors, including ILT2 and ILT4, as well as activating NKG2C and NKG2E on NK and T cells (Allan et al., 2002; Kaiser et al., 2005). These receptor engagements modulate cellular responses in contexts such as infection, cancer, transplantation, and pregnancy, positioning HLA-E as a critical mediator of immune homeostasis.

### 4.2 Role of HLA-E in IVF and reproductive outcomes

Although less extensively studied than HLA-G, HLA-E has emerged as a relevant immunogenetic factor in reproductive medicine, particularly in the context of IVF and RIF.

#### 4.2.1 Expression and functional role in the reproductive tract

HLA-E is expressed in decidual stromal cells, endometrial epithelial cells, and extravillous trophoblasts, where it contributes to immune tolerance and vascular remodeling during implantation. Studies have demonstrated that dysregulated HLA-E expression particularly in the endometrium may disrupt the local immune milieu, impairing receptivity and increasing the risk of implantation failure. Amjadi et al. (2020) observed reduced HLA-E expression in endometrial samples from women with RIF, alongside elevated levels of pro-inflammatory cytokines (e.g., Interleukin-6 (IL-6), Interferon-gamma (IFN-γ)) (Amjadi et al., 2020). This shift toward a Th1-dominant immune environment may prevent successful embryo implantation by limiting immune tolerance and promoting cytotoxic responses.

#### 4.2.2 HLA-E polymorphisms and fertility outcomes

Genetic studies have explored the association between HLA-E allelic variants and fertility. The two main coding alleles, HLA-E*01:01 and HLA-E*01:03, differ in their cell surface expression and peptide-binding affinity. Notably.

- HLA-E*01:03 is associated with higher surface stability and enhanced NK receptor binding, promoting stronger immunoregulatory effects.
- HLA-E*01:01, by contrast, shows reduced surface expression, which may compromise tolerance mechanisms.

A study of Euro-Brazilian couples undergoing ART found that HLA-E*01:03 homozygosity was more common among male partners of infertile couples compared to fertile controls, suggesting a potential role in male subfertility (Gelmini et al., 2016). Interestingly, no significant associations were observed among women in this cohort, emphasizing the importance of evaluating both male and female immunogenetic profiles in reproductive studies.

Other findings indicate possible ethnic variability in how HLA-E alleles affect reproductive outcomes. For instance, Tripathi et al. (2006) reported a positive association between HLA-E*01:01 and RM among Indian women (Tripathi et al., 2006), whereas studies from Japan and Denmark found no significant correlations (Steffensen et al., 1998; Kanai et al., 2001).

Across these studies, two patterns emerge. First, a male-specific signal has been reported in Euro-Brazilian ART couples, HLA-E*01:03 homozygosity was more common among male partners of infertile couples, while no significant association was detected among women in the same cohort, suggesting a possible contribution of HLA-E to male subfertility that requires replication in other populations (Gelmini et al., 2016). Second, ethnic variability is evident: an association between HLA-E*01:01 and RM has been reported in Indian cohorts, whereas studies from Japan and Denmark found no significant correlations, indicating that background allele frequencies, genetic architecture, and study design likely influence observed effects (Tripathi et al., 2006; Steffensen et al., 1998; Kanai et al., 2001).

Therefore, a comprehensive stratified meta-analysis considering sex, ethnicity, and infertility subtype is crucial to clarify HLA-E’s role in reproductive outcomes.

#### 4.2.3 Clinical implications and future directions

HLA-E’s ability to modulate NK cell and T cell responses at the maternal–fetal interface suggests it may influence multiple stages of reproduction, from implantation to placental development and pregnancy maintenance. Its interaction with NKG2A, in particular, may be critical for fine-tuning uNK cell activity, ensuring vascular remodeling without excessive cytotoxicity.

Recent advances in single-cell sequencing and immunophenotyping have enabled detailed mapping of HLA-E+ cells in the decidua and endometrium, revealing distinct expression patterns in women with successful versus failed implantation. Meanwhile, functional assays are beginning to explore how HLA-E polymorphisms alter NK receptor signaling, providing mechanistic insight into individual variability in IVF outcomes.

Although current evidence remains mixed and largely observational, HLA-E represents a promising target for.

- Immunogenetic screening of IVF patients.
- Embryo selection based on HLA-E expression in preimplantation embryos.
- Personalized immunotherapy strategies for RIF and RM.

Prospective, large, multi-ethnic cohorts with sex-stratified (maternal and paternal) genotyping and harmonized RM/RIF phenotypes, coupled with functional assays of HLA-E/CD94–NKG2A and downstream NK/T-cell responses, are needed to determine whether the observed male-specific and population-specific signals reflect true biology or study design.

## 5 HLA-F and its ligands in IVF

### 5.1 Structure, expression, and receptor interactions

HLA-F is a nonclassical HLA class I molecule first identified by Geraghty et al., in 1990. Structurally similar to classical HLA class I molecules, HLA-F comprises a ∼40–41 kDa α heavy chain paired with β2-microglobulin. However, HLA-F is distinct due to its alternative splicing, which often excludes exon 7, leading to a truncated cytoplasmic tail. This structural variation may affect intracellular trafficking and receptor signaling (Geraghty et al., 1990; Boyle et al., 2006) (Figure 1).

As of September 2025, the IPD-IMGT/HLA Database reports 126 HLA-F alleles, including 28 protein-coding and three non-functional (null) variants (Robinson et al., 2024), underscoring the relatively low polymorphism characteristic of nonclassical HLA genes.

HLA-F is expressed in a variety of immune cells including B cells, T cells, NK cells, and peripheral blood lymphocytes—and is inducible in activated immune environments. Its expression is predominantly intracellular under resting conditions, but surface expression is observed upon cell activation, suggesting immune-state–dependent regulation (Lee et al., 2010). In the reproductive tract, Shobu et al. (2006) found minimal surface expression of HLA-F on extravillous trophoblasts (EVTs) during the first trimester (Shobu et al., 2006), though Hackmon et al. (2017) later demonstrated increased HLA-F expression on migrating and invasive EVTs, pointing to a functional role during trophoblast invasion and early placentation (Hackmon et al., 2017).

One of the unique immunological features of HLA-F is its ability to exist in an open conformer state lacking a bound peptide, which enables it to interact with various immune receptors independently of antigen presentation. It binds a wide spectrum of inhibitory and activating receptors, including.

- ILT2 (LILRB1) and ILT4 (LILRB2)
- KIRs: KIR3DL2, KIR3DS1, and KIR2DS4 (Apps et al., 2008; Goodridge et al., 2013).

These receptor-ligand interactions enable HLA-F to modulate NK cell function, antigen-presenting cell activity, and overall immune tolerance at the maternal–fetal interface. HLA-F engagement with LILRB1 and LILRB2 on decidual macrophages and dendritic cells promotes a tolerogenic immune profile, facilitating trophoblast invasion, vascular remodeling, and immune quiescence, all of which are crucial during early pregnancy (Gardiner, 2008; Ishigami et al., 2015).

### 5.2 HLA-F in reproductive immunology and IVF

Recent studies have begun to shed light on the functional relevance of HLA-F in reproductive immunology and its potential contribution to IVF outcomes. While not as extensively studied as HLA-G or HLA-E, HLA-F is increasingly recognized as a key regulator of endometrial receptivity and immune tolerance.

#### 5.2.1 Expression during implantation

HLA-F is expressed in the endometrial epithelium and stromal cells, particularly during the mid-secretory phase, aligning with the “window of implantation”. Studies by Kofod et al. (2017) and Langkilde et al. (2020) demonstrated that HLA-F expression correlates with the abundance of CD56^+^ uNK cells, which are critical for establishing a supportive immune and vascular environment for implantation (Kofod et al., 2017; Langkilde et al., 2020). Higher HLA-F expression during this phase has been associated with improved implantation rates and clinical pregnancy outcomes following IVF (Mika et al., 2018). Conversely, low HLA-F expression in the endometrium has been linked to implantation failure and subfertility, likely due to insufficient immune regulation at the maternal–fetal interface. However, while Mika et al.‘s (Mika et al., 2018) identification of a GATA2-responsive enhancer region for HLA-F suggests transcriptional regulation under hormonal or immunological stimuli, the precise functional impact of these regulatory elements in endometrial biology remains unverified.

At the receptor level, in its peptide-free state, HLA-F binds KIR3DS1 and KIR3DL2 on NK cells, positioning it to tune uNK activation thresholds at the decidua (García-Beltrán et al., 2016). These receptor-mediated signals bias uNK toward a low-cytotoxic, pro-angiogenic phenotype, characterized by GM-CSF and VEGF secretion that enhances extravillous trophoblast invasion and spiral-artery remodeling (Xiong et al., 2013).

Additionally, although Burrows et al. (2016) identified HLA-F as a fecundability-associated gene in endometrial eQTL studies, direct *in vivo* validation linking HLA-F expression to functional NK cell modulation or endometrial receptivity is still lacking. These findings highlight the need for future studies that integrate molecular, immunological, and clinical approaches to fully clarify HLA-F’s mechanistic role in reproductive success.

#### 5.2.2 Genetic variants and IVF outcomes

Polymorphisms in the HLA-F gene, particularly single nucleotide polymorphisms (SNPs), have been correlated with variable expression and reproductive outcomes.

- The rs2523393 AA genotype has been associated with higher HLA-F expression, enhanced immune tolerance, and shorter time to pregnancy (Burrows et al., 2016; Mika et al., 2018).
- In contrast, RIF patients have shown higher frequencies of SNPs linked to reduced HLA-F mRNA and protein levels, suggesting that these variants may impair endometrial immune receptivity (Langkilde et al., 2020).

These findings suggest that HLA-F variants influence endometrial receptivity, potentially via uNK cell regulation. However, their clinical utility remains uncertain due to limited functional validation and population diversity.

#### 5.2.3 HLA-F in reproductive disorders

In a Danish cohort, Papúchová et al. (2022) reported abnormal HLA-F expression patterns in women with RIF, alongside signs of inflammation and hormonal dysregulation (Papúchová et al., 2022). Notably, women with polycystic ovary syndrome (PCOS) showed increased HLA-F expression but lower serum progesterone levels, suggesting that endocrine-immune crosstalk may influence HLA-F–mediated immune regulation and contribute to infertility. Although data remain limited, a recent systematic review by Han et al. (2023) highlighted two studies proposing a link between HLA-F polymorphisms and RIF, underscoring the need for broader research across diverse populations (Han et al., 2023). These findings suggest that HLA-F may exert differential effects depending on the underlying reproductive pathology, such as PCOS or RIF, and highlight the importance of hormone immune interactions in modulating its role in implantation.

#### 5.2.4 Clinical implications

HLA-F’s role in mediating maternal–fetal tolerance through NK cell and antigen-presenting cell interactions suggests it may be an important biomarker for endometrial receptivity and embryo implantation potential. Its expression pattern during the implantation window and genetic association with IVF outcomes offer exciting prospects for.

- Non-invasive endometrial profiling in IVF cycles
- Immunogenetic screening of patients with unexplained infertility or RIF
- Therapeutic interventions targeting HLA-F pathways to improve immune tolerance

#### 5.2.5 Conclusion

HLA-F is an emerging player in the field of reproductive immunology, contributing to immune homeostasis, implantation, and early pregnancy maintenance. Its capacity to regulate NK cell activity, promote immune tolerance, and respond to endocrine signals makes it a compelling target for both biomarker development and immunotherapeutic strategies in IVF. Future studies, particularly large-scale, multi-ethnic investigations, are needed to fully define the clinical relevance of HLA-F expression and polymorphisms. Integrating HLA-F analysis into personalized IVF protocols could enhance patient stratification and improve success rates, especially in those with repeated implantation failure or immune-mediated infertility.

## 6 MICA/B and their ligands in IVF

### 6.1 Genetic diversity and structure of MICA/B

MICA (MHC class I polypeptide-related sequence A) and MICB are nonclassical MHC class I molecules that exhibit extensive genetic diversity. As of September 2025, the IPD-IMGT/HLA Database lists 589 MICA alleles, including 29 protein-coding and 9 null variants, and 312 MICB alleles, with 53 protein-coding and 25 null variants (Robinson et al., 2024) (Figure 1). Unlike classical MHC class I molecules, MICA/B do not associate with β2-microglobulin, nor do they present peptides to T cells. Instead, they serve as stress-induced ligands for activating immune receptors.

Structurally, MICA/B are transmembrane glycoproteins composed of three extracellular α domains (α1, α2, α3), a transmembrane segment, and a cytoplasmic tail. Their promoter regions contain heat shock elements, making their expression highly inducible under cellular stress, including heat shock, oxidative damage, infection, and inflammation (Groh et al., 1999).

Under normal physiological conditions, MICA/B are expressed at low levels, predominantly in gut epithelium, thymus, endothelial cells, and fibroblasts. However, their expression is markedly upregulated during tumorigenesis, viral infection, autoimmune disease, and pregnancy-associated stress (Diefenbach and Raulet, 2002; Tieng et al., 2002). This dynamic regulation suggests a role for MIC molecules in immune surveillance and tissue adaptation during pregnancy.

### 6.2 MIC proteins and NKG2D-Mediated immune surveillance

The primary immunological role of MICA and MICB is their interaction with the NKG2D receptor, expressed on NK cells, CD8^+^ cytotoxic T lymphocytes (CTLs), and γδ T cells (Bauer et al., 2018). Unlike classical HLA-TCR recognition, the MIC–NKG2D axis enables MHC-unrestricted cytotoxicity, allowing immune cells to detect and eliminate stressed, infected, or transformed cells without antigen presentation (Diefenbach and Raulet, 2001).

MIC proteins exist in both membrane-bound and soluble forms. Soluble MICA/B (sMIC) are generated through proteolytic shedding by metalloproteinases, such as ADAM10 and ADAM17 (Waldhauer and Steinle, 2006). While membrane-bound MIC molecules activate immune effector functions, soluble MICs paradoxically inhibit immunity by downregulating NKG2D surface expression, leading to impaired NK and CD8^+^ T cell cytotoxicity (Groh et al., 2002; Doubrovina et al., 2003).

In the context of pregnancy, placental trophoblasts produce both membrane-bound and microvesicle-associated forms of MICA/B, implicating these proteins in maternal–fetal immune regulation. Studies suggest that trophoblast-derived sMIC contributes to local immune tolerance, modulating NK cell activity to support fetal survival (Mincheva-Nilsson et al., 2006).

### 6.3 MIC expression and IVF outcomes

Growing evidence links aberrant MIC expression and sMIC shedding with adverse reproductive outcomes, particularly in ART such as IVF. A study by Porcu-Buisson et al. (2007) found elevated serum sMIC levels in women undergoing IVF, which were significantly associated with implantation failure and early miscarriage (Porcu-Buisson et al., 2007). In a subsequent prospective analysis, Haumonte et al. (2014) reported that 38% of infertile women exhibited detectable sMIC levels, which correlated negatively with clinical pregnancy rates (Haumonte et al., 2014). These findings support the notion that sMIC may serve as a noninvasive biomarker of endometrial receptivity and immune dysfunction in IVF.

Moreover, altered metalloproteinase activity a key driver of sMIC shedding has been observed in women with RIF, potentially contributing to overproduction of sMIC and inhibition of NKG2D-mediated cytotoxic responses (Shibahara et al., 2005). This imbalance may suppress IFN-γ secretion, compromising trophoblast invasion and spiral artery remodeling, both essential for early placental development. MIC shedding has also been implicated in autoimmune and alloimmune disorders, which frequently co-occur with infertility. Elevated sMIC levels have been reported in women with celiac disease, autoimmune thyroiditis, and systemic lupus erythematosus conditions associated with increased risk of miscarriage and poor IVF outcomes (Meloni et al., 1999; Meresse et al., 2004). These findings suggest that sMIC levels reflect not only local uterine immunoregulation but also systemic immune activation.

In IVF patients undergoing preimplantation genetic testing (PGT), sMIC levels were found to be elevated in those who later developed intrauterine growth restriction (IUGR) or PE pregnancy complications often linked to vascular dysfunction and immune dysregulation (Basu et al., 2008; Raulet et al., 2013). These observations further implicate MIC molecules in vascular remodeling, decidualization, and placental homeostasis. Overall, evidence links elevated sMIC with impaired implantation and placental development, but inconsistencies in assay methods and patient selection highlight the need for standardized protocols and longitudinal studies to confirm its predictive value in IVF.

### 6.4 Conclusion and future directions

Collectively, the MICA/B–NKG2D axis represents a pivotal immune checkpoint in reproductive immunology. MIC proteins, by serving as stress-inducible ligands for activating immune receptors, participate in both immune surveillance and immune evasion, depending on their form (membrane-bound vs. soluble) and regulatory context.

In the setting of IVF, dysregulated MIC expression whether through genetic variation, inflammatory stress, or aberrant shedding may impair NK and T cell function, contributing to implantation failure, miscarriage, and placental pathologies such as PE and IUGR. Key Areas for Future Investigation Include.

- Mechanisms of sMIC generation and regulation in early pregnancy and ART settings.
- MICA/B genetic polymorphisms and their associations with implantation success, RIF, and live birth rates.
- Utility of serum sMIC levels as a predictive biomarker for endometrial receptivity and pregnancy outcome in IVF cycles.

As research advances, targeting the MIC–NKG2D pathway may offer novel therapeutic strategies. For example, metalloproteinase inhibitors could prevent excessive shedding of MIC molecules from the cell surface, thereby preserving their interaction with activating immune receptors. NKG2D agonists may enhance NK cell and cytotoxic T cell recognition of abnormal cells, while MIC-stabilizing agents could prolong surface expression and maintain effective immune surveillance may help rebalance immune tolerance and improve ART success. Integrating MIC profiling into personalized IVF protocols could enhance embryo selection, predict implantation potential, and improve outcomes for patients with unexplained infertility or repeated IVF failure.

## 7 Debate on testing for non-classical HLA genes prior to IVF

Non-classical HLA class I molecules including HLA-G, HLA-E, HLA-F, and MICA/B play indispensable roles in establishing maternal–fetal immune tolerance, particularly during implantation and early placentation. Unlike classical HLA class I molecules involved in antigen presentation, these molecules are predominantly immunomodulatory, acting to suppress excessive immune responses at the maternal–fetal interface. Given the growing recognition of immune dysregulation as a contributor to implantation failure, RM and pregnancy complications, there is rising interest in evaluating whether pre-implantation genetic screening of non-classical HLA genes could enhance outcomes in IVF and related ART.

### 7.1 Arguments in favor of non-classical HLA testing

Supporters of pre-implantation HLA immunogenetic screening posit that identifying maternal and paternal polymorphisms in non-classical HLA genes may reveal immunogenetic mismatches that predispose patients to implantation failure, PE, or recurrent pregnancy loss (RPL).

One well-studied example involves maternal KIR haplotypes and their interaction with fetal HLA-C ligands. Several studies have shown that the KIR AA haplotype, which lacks activating KIRs, is associated with higher risk of early miscarriage, placental insufficiency, and lower live birth rates, particularly in oocyte donation cycles where maternal–fetal HLA compatibility may be further reduced (Alecsandru et al., 2014; Zhang and Wei, 2021). While promising, KIR/HLA-C testing remains limited by inconsistent predictive power across ethnic groups, underscoring the need for population-specific validation before routine clinical use.

Moreover, soluble HLA-G (sHLA-G) levels along with specific HLA-G 3′UTR polymorphisms (e.g., the 14-bp ins/del variant) have been linked to implantation potential and pregnancy maintenance. A study by Alecsandru et al. (2020) suggests that low sHLA-G expression in embryo culture media, especially in combination with specific maternal genotypes, may correlate with poorer IVF outcomes (Alecsandru et al., 2020).

Emerging research has also identified associations between HLA-F SNPs (e.g., rs2523393) and improved endometrial receptivity, as well as HLA-E*01:03 homozygosity and impaired male fertility, suggesting a broader role for non-classical HLA polymorphisms across both partners in influencing reproductive success (Gelmini et al., 2016; Mika et al., 2018; Langkilde et al., 2020). Yet, most findings come from small-scale or single-center studies, making replication in larger, multi-ethnic cohorts essential to determine true clinical utility.

### 7.2 Technological advances supporting functional interpretation

Recent advances in machine learning and bioinformatics have significantly enhanced our ability to predict the functional consequences of non-classical HLA polymorphisms. For instance, DeepHLAPred, a deep learning-based tool, can accurately forecast ligand-binding affinity for both classical and non-classical HLA variants, offering insights into immune receptor engagement and signaling potential (Huang et al., 2023). Such tools may soon enable *in silico* screening of HLA genotypes prior to embryo transfer, identifying high-risk immunogenetic profiles and guiding immunomodulatory interventions.

In parallel, innovations in single-cell RNA sequencing, decidual immune profiling, and AI-assisted embryo selection are facilitating a more integrated approach to evaluating maternal–fetal immunological compatibility. These technologies promise to shift IVF from a one-size-fits-all approach toward precision reproductive immunology.

### 7.3 Challenges and counterarguments

Despite these advancements, the routine clinical application of non-classical HLA genotyping in IVF remains controversial for several reasons.

#### 7.3.1 Lack of standardized testing protocols

Currently, there are no validated, consensus-driven clinical guidelines for testing HLA-E, HLA-F, or MICA/B polymorphisms in reproductive settings. Testing platforms vary in their sensitivity, specificity, and interpretive algorithms.

#### 7.3.2 Limited clinical validation

Most studies linking non-classical HLA polymorphisms to IVF outcomes have small sample sizes, retrospective designs, and limited ethnic diversity. No large, prospective, multicenter trials have yet confirmed the clinical utility of such testing in improving live birth rates or reducing miscarriage risk.

#### 7.3.3 Cost and accessibility

High-throughput HLA sequencing technologies are expensive and not widely available, especially for non-classical HLA loci, which are often excluded from standard HLA panels used in transplantation or immunogenetic labs.

#### 7.3.4 Complexity of implantation biology

Implantation success depends on multiple intersecting variables, including cytokine profiles, hormonal balance (e.g., progesterone levels), endometrial receptivity markers (e.g., Leukemia Inhibitory Factor (LIF), integrins), and embryo quality. HLA compatibility is only one piece of a multifactorial puzzle, and over-reliance on HLA testing could lead to overmedicalization without clear clinical benefit.

### 7.4 Conclusion and future perspectives

While the concept of non-classical HLA genotyping prior to IVF is scientifically compelling, current evidence is insufficient to recommend routine clinical implementation. Nonetheless, ongoing research is rapidly expanding our understanding of how HLA-G, HLA-E, HLA-F, and MICA/B shape maternal immune responses during early pregnancy. Future large-scale, ethnically diverse, and functionally validated studies are essential to determine.

- Which polymorphisms or haplotypes are truly predictive of implantation failure or adverse pregnancy outcomes.
- How maternal–paternal HLA combinations influence immune compatibility and fetal development.
- Whether targeted immunomodulatory therapies based on HLA genotyping can meaningfully improve IVF success.

If these questions are answered affirmatively, non-classical HLA testing may become a cornerstone of personalized IVF protocols, guiding embryo transfer strategies, immune therapies, and patient counseling in cases of unexplained infertility or RIF.

## 8 Conclusion and future directions

Non-classical HLA class I molecules including HLA-G, HLA-E, HLA-F, and the MICA/B family—are increasingly recognized as central regulators of maternal–fetal immune tolerance, particularly during embryo implantation, early placentation, and pregnancy maintenance. These molecules interact with a diverse array of immune receptors on NK cells, cytotoxic CD8^+^ T cells, γδ T cells, and antigen-presenting cells (APCs), shaping the local immunological landscape at the maternal–fetal interface.

Among them, HLA-G has been the most extensively studied. Its soluble isoform (sHLA-G) has shown promise as a biomarker for embryo viability, correlating with improved implantation rates and early pregnancy success in IVF. However, newer studies have illuminated the distinct and non-redundant roles of HLA-E, HLA-F, and MICA/B, which contribute to reproductive outcomes via their expression profiles, receptor-binding affinities, and polymorphic variations.

### 8.1 Emerging insights: MICA/B and immune regulation in IVF

In particular, MICA and MICB stress-inducible ligands that bind to the NKG2D receptor demonstrate a dual immunological function. Membrane-bound MICA/B enhance immune activation in response to cellular stress, while soluble forms (sMIC), generated by proteolytic shedding, may downregulate NKG2D and suppress cytotoxic immune responses. This balance is crucial during implantation, where excessive immune activation or suppression can impair endometrial receptivity. Recent research has linked elevated sMIC levels to.

- Impaired implantation due to decreased NKG2D expression on uNK cells and CD8^+^ T cells.
- Increased risk of miscarriage, potentially signaling alloimmune or inflammatory imbalance.
- Complications such as PE and IUGR likely resulting from defective trophoblast invasion and spiral artery remodeling.

Further, polymorphisms such as MICA*008, promoter variants, and regulatory SNPs have been associated with differences in sMIC shedding, NKG2D affinity, and reproductive outcomes. However, the clinical application of MIC genotyping remains limited due to population variability, lack of assay standardization, and incomplete mechanistic data.

### 8.2 Key priorities for future research

To realize the full clinical value of non-classical HLA and MIC molecules in reproductive medicine, future research should focus on the following critical areas.

#### 8.2.1 Multinational, multi-ethnic cohort studies

- Validate associations between non-classical HLA/MIC polymorphisms and implantation, miscarriage, and live birth outcomes across diverse populations.
- Incorporate both maternal and paternal immunogenetic profiles to capture the full scope of maternal–fetal compatibility.

#### 8.2.2 Functional mechanistic studies

- Elucidate the biological impact of HLA-G, HLA-E, HLA-F, and MICA/B variants on NK cell function, cytokine production, and trophoblast invasion.
- Investigate the role of sMIC generation and NKG2D receptor modulation during the peri-implantation window.

#### 8.2.3 Predictive and diagnostic tool development

- Develop AI-assisted platforms (e.g., DeepHLAPred) capable of predicting HLA-receptor interactions, immune risk stratification, and embryo selection outcomes.
- Integrate sHLA-G and sMIC measurements into IVF laboratory workflows to inform embryo transfer timing and immunomodulatory treatment decisions.

#### 8.2.4 Translational trials of targeted immunotherapies

- Evaluate checkpoint modulators, cytokine inhibitors, or metalloproteinase inhibitors to restore immune homeostasis in patients with known HLA/KIR incompatibilities or sMIC overexpression.
- Explore personalized immunomodulation in patients with RIF or immunologically mediated infertility.

## 9 Translational potential and implementation challenges

The integration of non-classical HLA and MIC gene profiling into reproductive medicine offers considerable promise for improving implantation outcomes, reducing pregnancy complications, and tailoring IVF strategies to individual immunogenetic profiles. These molecules through their influence on immune tolerance, vascular adaptation, and embryo–maternal compatibility could serve as valuable biomarkers and therapeutic targets in precision fertility care.

However, moving from research to routine clinical use requires overcoming several key challenges.

- Evidence Standardization and Validation

Current findings are often based on small or heterogeneous cohorts, with variable study designs and inconsistent outcome measures. Large-scale, multi-ethnic, and sex-stratified clinical trials are needed to confirm predictive value and generalizability.

- Assay Development and Accessibility

Reliable, cost-effective, and reproducible genotyping or expression assays must be developed and validated for use in IVF laboratories. Harmonizing protocols across centers is essential to ensure comparability of results.

- Integration into Clinical Workflows

Implementation will require minimal disruption to established IVF timelines and procedures. This includes compatibility with embryo selection methods, laboratory infrastructure, and patient counseling processes.

- Interpretation and Decision-Making

Clear clinical guidelines are needed to translate immunogenetic findings into actionable recommendations, avoiding overinterpretation of associations without functional validation.

- Ethical and Regulatory Considerations

Incorporating HLA and MIC testing into embryo selection raises questions about informed consent, data privacy, and the ethical implications of selecting for specific genetic profiles.

- By addressing these practical considerations alongside scientific discovery, the field can move toward responsible clinical adoption. Strategic collaboration between reproductive specialists, immunologists, molecular biologists, and bioinformaticians will be critical in bridging the gap between promising molecular insights and real-world patient benefit.

## 10 Conclusion

As reproductive medicine shifts toward precision care, the integration of non-classical HLA and MIC gene analysis offers a promising avenue to enhance implantation success, reduce pregnancy complications, and individualize treatment strategies in IVF. Their utility spans biomarker discovery, immune compatibility screening, and targeted intervention development, highlighting the importance of interdisciplinary collaboration among reproductive specialists, immunologists, molecular biologists, and computational scientists.

Ultimately, the clinical translation of these findings will depend on robust scientific validation, scalable technological platforms, and a systems-level understanding of reproductive immunology. By embracing this complexity, we move closer to a future of immunologically informed, patient-centered fertility care one that optimizes outcomes for individuals and couples navigating the challenges of infertility.
